# Supplementary material for: Pattern of seasonal variation in rates of predation between spider families is temporally stable in a food web with widespread intraguild predation
Source: PLoS One. 2023 Oct 30;18(10):e0293176. doi: 10.1371/journal.pone.0293176 (PMC10615273; doi:10.1371/journal.pone.0293176)
Supplement: S1 File — (DOCX) [file pone.0293176.s001.docx]

**S1 File: Rates of prey detection**

Of 3299 spiders analyzed (3300 minus the possibly anomalous thomisid), 1,467 tested positive for one prey item, 17 tested positive for two prey taxa, and none tested positive for ≥ 3 prey. If the probabilities of detecting 0, 1, 2, 3 . . . prey taxa in one spider are independent, one would expect 7.5% of the spiders analyzed to test positive for two or more prey (fitting a Poisson process with λ = 0.45 to our data). In our data set, only 17 (0.5%), tested positive for ≥ 2 prey. Given our large sample size, prey detection clearly was not a simple Poisson process. What might be causing this deviation from independence i.e., why does the probability of detecting DNA of a second prey taxon depend upon the presence of another taxon in the spider gut? Several possible explanations come to mind that are related to spider ecology, our collecting protocol, and possible limitations of multiplex PCR. The most likely explanations are the following:

- Spider populations often are food limited and direct observations have revealed that spiders often may consume only one prey item per day (1).
- Adult males in many spider families do not feed as frequently as adult females (2). Over half (55%; S1 Table) of spiders we analyzed were adult males.

------The above two factors suggest that many spiders likely had eaten only one prey item in the previous day or two. A higher rate of predation could have increased the chances of detecting multiple types of prey DNA before it had broken down into fragments too small to be detected by the primers utilized in our multiplex PCR analysis (King et al. 2008).

*(Note: one might argue that the contribution of the above two factors is directly reflected by the number of “failures” (spiders in which prey was not detected) used to fit the Poisson distribution to the observed data. If we were to accept this argument, we would still need to explain why multiplex PCR failed to detect the expected number of multiple predation events.)*

- The 93 collecting days were distributed across three seasons in each of four years. Not all potential prey would have been consistently nor equally represented on each collecting day. Thus, a spider would appear to be more specialized, in comparison with its entire prey spectrum across the entire study, when observed at only one of these 93 time points. Hence, in our study the rate of prey detection used to fit a Poisson is a highly heterogeneous parameter, which complicates interpreting the deviation from a fitted Poisson.
- The ability of multiplex PCR to detect several different prey items in a single predator sample depends upon many factors (3). We necessarily employed multiplex PCR to analyze the prey of 3300 spiders because 21 possible predator-prey interactions needed to be tested for each spider. Testing the effectiveness and sensitivity of our multiplex procedures for all combinations of prey and predators would have been unfeasible. Although our multiplex protocol did detect more than one prey item in several samples, it may not have been optimal for the wide range of potential prey for which we tested.

In conclusion: Given the high diversity of spiders and potential prey, the extended collecting period, and the challenges of multiplex PCR, the low number of spiders for which two prey was detected is not as surprising as might first appear and is no cause for concern. Reliance on multiplex PCR was the only feasible way to examine each of 3300 spiders for 21 possible trophic interactions. Even if multiplex PCR had failed to uncover some joint prey occurrences, there is no reason to question its overall effectiveness in uncovering broad patterns of trophic interactions, as our prey detection rate is comparable to that of other studies. Following are two examples of the application of primer-based singleplex PCR to similar systems. In a study of predation by 1,231 spiders in one genus of Lycosidae in a North American forest, Whitney et al. (4) found that 44% tested positive for Collembola and 33% tested positive for Diptera. In a study of 128 spiders (representing 11 families) from two Russian forests, Zuev et al. (5) found that 17% of 968 possible interactions (128 x 6 different non-spider prey) tested positive for prey DNA.

References

1. Wise DH. Spiders in Ecological Webs. Cambridge: Cambridge University Press; 1993. 328 p.

2. Foelix RF. Biology of Spiders. Second ed. Oxford: Oxford Univeristy Press; 2011. 432 p.

3. King RA, Read DS, Traugott M, Symondson WOC. Molecular analysis of predation: a review of best practice for DNA-based approaches. Molecular Ecology. 2008;17(4):947-63.

4. Whitney TD, Sitvarin MI, Roualdes EA, Bonner SJ, Harwood JD. Selectivity underlies the dissociation between seasonal prey availability and prey consumption in a generalist predator. Molecular Ecology. 2018;27(7):1739-48.

5. Zuev A, Heidemann K, Leonov V, Schaefer I, Scheu S, Tanasevitch A, et al. Different groups of ground-dwelling spiders share similar trophic niches in temperate forests. Ecological Entomology. 2020;45(6):1346-56.
