# Supplementary material for: Pattern of seasonal variation in rates of predation between spider families is temporally stable in a food web with widespread intraguild predation
Source: PLoS One. 2023 Oct 30;18(10):e0293176. doi: 10.1371/journal.pone.0293176 (PMC10615273; doi:10.1371/journal.pone.0293176)
Supplement: S1 Table — (PDF) [file pone.0293176.s002.pdf]

S1 Table. Number of spiders per genus analyzed, and testing positive, for prey DNA. Spiders were selected at random from the most abundant genera to yield 25, 50, 75, 100, or 150 per genus (roughly in proportion to relative abundances) to yield 300 / family. Only adults (0.56:0.44::male:female (total no. positive)) were analyzed.

Family-level Foraging Mode	**Family**/Genus	No. Tested for Prey DNA	No. positive for Prey DNA	Percent Positive
Web Spinners	**Agelenidae**	**300**	**112**	**37%**
	Agelenopsis	300	112	37%
	**Theridiidae**	**300**	**110**	**37%**
	Enoplognatha	200	68	34%
	Robertus	100	42	42%
	**Hahniidae**	**300**	**138**	**46%**
	Hahnia	100	51	51%
	Neoantistea	200	87	44%
	**Dictynidae**	**300**	**145**	**48%**
	Argenna	100	52	52%
	Cicurina	100	47	47%
	Emblyna	100	46	46%
	**Linyphiidae**	**300**	**180**	**60%**
	Bathyphantes	50	33	66%
	Centromerus	50	26	52%
	Diplostyla	25	14	56%
	Eperigone	25	15	60%
	Meioneta	50	30	60%
	Microneta	50	37	74%
	Neriene	50	25	50%
Cursorial	**Thomisidae**	**300**	**155**	**52%**
	Bassaniana	50	35	70%
	Coriarachne	50	35	70%
	Ozyptila	100	45	45%
	Xysticus	100	41	41%
	**Pisauridae**	**300**	**113**	**38%**
	Dolomedes	100	39	39%
	Pisaurina	200	74	37%
	**Lycosidae**	**300**	**135**	**45%**
	Pardosa	75	38	51%
	Pirata	75	28	37%
	Schizocosa	75	33	44%
	Trochosa	75	36	48%
	**Gnaphosidae**	**300**	**124**	**41%**
	Drassyllus	100	47	47%
	Sergiolus	100	43	43%
	Sosticus	100	34	34%
	**Salticidae**	**300**	**140**	**47%**
	Habrocestrum	100	52	52%
	Maevia	50	12	24%
	Neon	100	47	47%
	Pelegrina	50	29	58%
	**Corinnidae**	**300**	**123**	**41%**
	Castianeira	150	63	42%
	Phrurotimpus	150	60	40%
