# Supplementary material for: Pattern of seasonal variation in rates of predation between spider families is temporally stable in a food web with widespread intraguild predation
Source: PLoS One. 2023 Oct 30;18(10):e0293176. doi: 10.1371/journal.pone.0293176 (PMC10615273; doi:10.1371/journal.pone.0293176)
Supplement: S2 Table — (PDF) [file pone.0293176.s003.pdf]

S2 Table. A more detailed analysis of patterns in the summary web. A few results given below also appear in the main text; they are included here for completeness.

Summary Bullet Points	Description
(i)	The three most-commonly consumed non-spider taxa (Diptera, Lepidoptera and Collembola) appear in the diets of 6-9 spider families. All five web-spinning families consumed Diptera and Lepidoptera, whereas only 3-4 cursorial spiders fed on these taxa. Most cursorial spiders (5/6 families), but only one web spinner (Linyphiidae), fed on Collembola.
(ii)	The eight less-commonly consumed non-spider taxa appeared almost exclusively in the diets of cursorial spiders. Two web-spinning families fed on Gryllidae or Pseudoscorpiones, but rarely.
(iii)	IGP is widespread in this food web, as slightly less than 50% of the prey consumed by spiders were other spiders.
(vi)	All spiders except Agelenidae appeared in the diets of 2-6 other spider families; no spiders fed on agelenids.
(v)	IGP was largely confined within the two broadly defined foraging modes of cursorial and web-spinning spiders; 90.5% (66/698) of all IGP involved other families within the same foraging type. Cursorial spiders fed primarily on other cursorial spiders, because web spinners accounted for 7.2% (22/306) of cursorial IGP prey. Web spinners preyed largely on other web spinners, as 11.2% (44/392) of their IGP prey were cursorial spiders.
(vi)	Linyphiids (web spinners) formed a substantial fraction of the diets of three other web-spinning families (Hahniidae, Theridiidae, and Dictynidae).
(vii)	Other spiders comprised over 50-75% of the diets of four web-spinning families; among web spinners, only agelenids rarely fed on other spiders.
(viii)	Other spiders also comprised a substantial fraction (~50%) of the diets of five families of cursorial spiders; among cursorial families only pisaurids preyed primarily upon non-spider taxa.
