# Supplementary material for: Pattern of seasonal variation in rates of predation between spider families is temporally stable in a food web with widespread intraguild predation
Source: PLoS One. 2023 Oct 30;18(10):e0293176. doi: 10.1371/journal.pone.0293176 (PMC10615273; doi:10.1371/journal.pone.0293176)
Supplement: S3 Table — (PDF) [file pone.0293176.s004.pdf]

S3 Table. Comparison of seasonal shifts in % IGPrey and seasonal shifts in interaction pathways. Predation pathways are detailed in (A) summary web, (B) spring and fall webs pooled, and (C) summer web; the KEY appears after the summary web. Structure of the interaction matrices is explained in Section 2.4.1 of the text. Full names of the spider families and non-spider prey taxa appear Section 2.2 of the text. Spring and fall webs were pooled because sample sizes were smaller than the summer web; and equally important, because % IGPrey in spring and fall webs were similar (refer to texts for details). To the right of the interaction summary for each web is a summary of the different types of interaction pathways. (D) Patterns in different ways of looking at frequencies of IGPrey. It is clear that seasonal changes in % IGPrey among all prey (the index presented in the text) are not due to changes in the relative number of IGP modules in the web. In fact, the pattern of distribution of interaction pathways is remarkably similar for summary, spring+fall, and summer food webs. Thus, seasonal changes in % IGPrey reflect changes in the distribution of interactions within persistent IGP modules.

(A) SUMMARY

	TOTAL	LYCO	PISA	THOM	GNAP	SALT	CORI	LINY	HAHN	THER	DICT	AGEL	DIP	LEPI	COLL	HYME	ARAC	GRYL	PSEU	OPIL	COLE	DERM	ISOP
Lyco	139		6	12	9	12		7					14		14		12	15	2	13	14	7	**2***
Pisa	113	10		4						3			61	23						12			
Thom	155	38	20			7							22	10	25		22		11				
Gnap	124	37		4			3	9					10	5	12	35					9		
Salt	140	29	15	16			4						8		12	7	24	17	6			2	
Cori	125	22	3	17	16				3						27	37							
Liny	182	8	10						31	28	22		36	14	33								
Hahn	141			9				98					15	19									
Ther	110		8					49	17		2		17	9					**8***				
Dict	147							68	22	11			34	12									
Agel	112		9										56	32				**15***					
	1488																						

**Predation on SPIDERS:**		
**IGP Modules**		**37**
Reciprocal IGP	33	
Asymmetrical IGP	4	
**Non-IGP interactions**		**2**
**Total**		**39**
**Predation on NON-SPIDERS:**		
**IGP Modules**		**41**
**Non-IGP interactions**		**3**
**Total**		**44**

KEY: n **RECIPROCAL IGP** (white) · n **Asymmetrical (NON-RECIPROCAL) IGP** (blue) · n **NO IGP: no shared resource** (red) · n **Non-spider prey** (grey) · **n*** **Non-IGP interaction** · **No Interaction** (empty) · **Differs from SUMMARY Web** (yellow)

(B) Spr + Fall

	TOTAL	LYCO	PISA	THOM	GNAP	SALT	CORI	LINY	HAHN	THER	DICT	AGEL	DIP	LEPI	COLL	HYME	ARAC	GRYL	PSEU	OPIL	COLE	DERM	ISOP
Lyco	72		4	3	4	4		2					7		10		8	10		8	8	3	**1***
Pisa	51	1								2			30	12						6			
Thom	96	13	10			1							16	8	18		20		10				
Gnap	83	23					2	3					7	4	9	27					8		
Salt	93	13	11	7			3						7		7	7	15	16	6			1	
Cori	83	5	2	7	3				3						26	37							
Liny	131	3	9						18	16	13		33	10	29								
Hahn	79			3				48					11	17									
Ther	65		5					24	7				14	7					**8***				
Dict	71							36	4	3			18	10									
Agel	89		9										47	33									
	913																						

**Predation on SPIDERS:**		
**IGP Modules**		**35**
Reciprocal IGP	32	
Asymmetrical IGP	3	
**Non-IGP paths**		**2**
**Total**		**37**
**Predation on NON-SPIDERS:**		
**IGP Modules**		**40**
**Non-IGP paths**		**2**
**Total**		**42**

(C) Summer

	TOTAL	LYCO	PISA	THOM	GNAP	SALT	CORI	LINY	HAHN	THER	DICT	AGEL	DIP	LEPI	COLL	HYME	ARAC	GRYL	PSEU	OPIL	COLE	DERM	ISOP
Lyco	67		2	9	5	8		5					7		4		4	5	2	5	6	4	**1***
Pisa	62	9		4						1			31	11						6			
Thom	59	25	10			6							6	2	7		2		1				
Gnap	41	14		4			1	6					3	1	3	8					1		
Salt	47	16	4	9			1						1		5		9	1				1	
Cori	42	17	1	10	13										1								
Liny	51	5	1						13	12	9		3	4	4								
Hahn	62			6				50					4	2									
Ther	45		3					25	10		2		3	2									
Dict	76							32	18	8			16	2									
Agel	23												9					**14***					
	575																						

**Predation on SPIDERS:**		
**IGP Modules**		**36**
Reciprocal IGP	33	
Asymmetrical IGP	3	
**Non-IGP paths**		**1**
**Total**		**37**
**Predation on NON-SPIDERS:**		
**IGP Modules**		**37**
**Non-IGP paths**		**2**
**Total**		**39**

(D) Patterns in different ways of looking at frequencies of IGPrey

**WEB**	Predation on IGPrey	Total predation	**% IGPrey among all prey**	Predation on spiders	**% IGPrey among spider prey**	Predation on non-spider prey	Predation on non-spid prey in IGP modules
**SUMMARY**	692	1488	**46.50%**	698	**99.1 %** (692/698)	1488-692 = 796	771
**Spr + Fall**	319	913	**34.90%**	324	**98.5%** (319/324)	913-324 = 589	580
**Summer**	373	575	**65.00%**	374	**99.7%** (373/374)	575-374 = 201	196
